# Switching of Skyrmion chirality by local heating

**DOI:** 10.1038/s41598-019-49875-7

**Published:** 2019-09-17

**Authors:** Yoshinobu Nakatani, Keisuke Yamada, Atsufumi Hirohata

**Affiliations:** 1 Graduate school of Informatics and Engineering, University of Electro- Communications, Chohu, Tokyo Japan; 2 Department of Chemistry and Biomolecular Science, Faculty of Engineering, Gifu University, Gifu-shi, Gifu Japan; 3 Department of Electronic Engineering, University of York, York, United Kingdom

**Keywords:** Electronic devices, Magnetic devices

## Abstract

Magnetic Skyrmions are energetically stable entities formed in a ferromagnet with a diameter of typically below 100 nm and are easily displaceable using an electrical current of 10^2^ A/cm^2^, resulting the Skyrmions to be more advantageous than domain walls for spintronic memory applications. Here, we demonstrated switching of a chirality of magnetic Skyrmions formed in magnetic thin films by introducing a pulsed heat spot using micromagnetic simulation. Skyrmions are found to expand with a pulsed heat spot, which induces the magnetic moments surrounding the Skyrmion to rotate by this expansion, followed by the chirality switching of the Skyrmion. Such simple controllability can be used as a fundamental building block for memory and logic devices using the chirality of Skyrmions as a data bit.

## Introduction

A magnetic Skyrmion^1–5^ is a chiral structure formed in a magnetic thin film by the Dzyaloshinskii-Moriya interaction (DMI)^6,7^. A typical size of a Skyrmion is in the diameter between several tens and several hundreds of nanometers, and the threshold current density to move a Skyrmion is five orders of magnitude smaller than that for a conventional magnetic domain wall (DW). It is hence expected to be used as an information carrier for next-generation magnetic storage and logic systems reproducibly^8–10^. In order to implement Skyrmions into such device applications, it is required to control their nucleation and annihilation, and their degrees of freedom, *i.e*., the vorticity *V*, polarity *P* and helicity *H* (including chirality *C*)^11^. It is also crucial to precisely control the Skyrmion motion by introducing an external magnetic field and a spin-polarized current^8–10^. For the nucleation of a Skyrmion, several methods have been reported to be used such as the utilization of a sample edge^9,12^ and the application of a local external electromagnetic waves, *e.g*., a spin-current^13–15^, local thermal heating^16,17^ and an electric field^18^. It has been reported that the polarity of a Skyrmion can be controlled by applying electric fields in several nano-second pulses^18^. However, the vorticity and helicity of a Skyrmion are randomly appeared by introducing local thermal heating in several hundred nano-seconds^17^. However, it has not reached the stage to control the Skyrmion by local thermal heating.

The helicity of Skyrmion is one of the key degrees of freedom, which can be used as a memory bit or a logic data. In a material under DMI, a Skyrmion with the vorticity *V* = 1 and the helicity *H* = ± π/2, corresponding to the two chirality states *C* = ± 1, *i.e*., clockwise (CW) and counter clockwise (CCW) respectively, is an energetically stable structure^11,19^. The chirality is therefore a desirable parameter to be controlled for memory and logic operation as it can be changed by the DM vector, which is determined by the crystal structure. However there has been no study reported to date to switch the chirality of a Skyrmion with maintaining the same of DM vector for device applications.

In this paper, we propose a method to switch the chirality of a Skyrmion by applying a local pulsed heat spot, which has been demonstrated by our simulations. We have clarified the relationship between heat spot size and heat pulse length required for controlling the chirality of a Skyrmion. Furthermore, we have revealed that the mechanism of the heat-induced chirality switching, which is found to be dependent on the initial chirality state (either CW or CCW).

## Methods

A micromagnetic model was used to calculate the motion of the magnetic moments in a thin-film using the Landau-Lifshitz-Gilbert equation with the following form

1 $$\frac{\partial {\boldsymbol{m}}}{\partial t}=-|\gamma |{\boldsymbol{m}}\times {{\boldsymbol{H}}}^{{\boldsymbol{eff}}}+{\boldsymbol{\alpha }}m\times \frac{\partial {\boldsymbol{m}}}{\partial t}$$

Here, ***m***, ***H***^eff^, γ, and α are the unit magnetization vector that represents the direction of a local magnetic moment, the effective magnetic field acting on the magnetization, the gyromagnetic ratio, and the Gilbert damping constant, respectively^20,21^. The effective magnetic field is calculated from the magnetic energy density by ${{\boldsymbol{H}}}^{{\boldsymbol{eff}}}=-\frac{1}{{M}_{s}}\frac{\delta \varepsilon }{\delta {\boldsymbol{m}}}$. For the magnetic energy, the exchange, anisotropy, demagnetizing, and Dzyaloshinskii-Moriya exchange interaction energies are taken into account in the simulation^22^.

2 $$\begin{array}{c}\varepsilon =A{(\nabla {\boldsymbol{m}})}^{2}+{K}_{u}(1-{m}_{z}^{2})-\frac{1}{2}{M}_{s}{\boldsymbol{m}}\cdot {{\boldsymbol{H}}}^{dem}\\ +D[({m}_{x}\frac{\partial {m}_{z}}{\partial x}-{m}_{z}\frac{\partial {m}_{x}}{\partial x})+({m}_{y}\frac{\partial {m}_{z}}{\partial y}-{m}_{z}\frac{\partial {m}_{y}}{\partial y})]\end{array}$$

where, *A*, *K*_u_, *M*_s_, *H*^dem^, and *D* are the exchange stiffness constant, the uniaxial anisotropy constant, the saturation magnetization, the demagnetizing field, which is calculated numerically, and the DM exchange constant, respectively. Here we assumed the interfacial DMI between the ferromagnetic thin film and the non-magnetic layer underneath. Our thin film model possessed dimensions of 256 × 256 × 1.4 nm^3^, and was further divided into rectangular prisms with their dimensions of 0.5 × 0.5 × 1.4 nm^3^. Free boundary condition was used at the disk edge^22^.

3 $$\begin{array}{ll}\frac{d\theta }{dn}=\frac{1}{\xi }, & \xi =\frac{2A}{D}\end{array}$$

Here *θ* is a polar angle of a magnetic moment, *n* is the normal direction at the disk edge, and ξ is a characteristic length determined by *D*^22^. Typical material parameters for perpendicularly-magnetized CoFeB thin films at room temperature (300 K) were used, *i.e*., a saturation magnetization *M*_s_ = 1600 emu/cm^3^, an exchange stiffness constant *A* = 3.1  × 10^−6^ erg/cm, an uniaxial anisotropy constant *K*_u_ = 16.2 Merg/cm^3^, a gyromagnetic ratio γ = 1.76  × 10^7^ rad/(s · Oe), the Gilbert damping constant *α* = 0.1, and the DMI constant *D* = 0.6 erg/cm^2^ ^3,23–25^.

**Figure 1:**
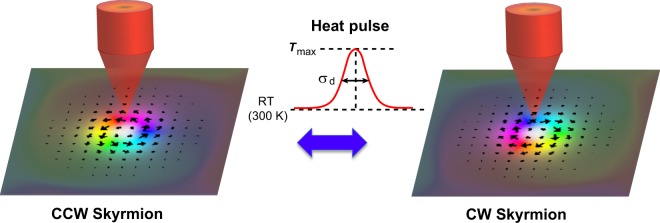
Schematic illustration of the system under study. A pulsed heat spot is applied to the film.

Two types of Skyrmions with CCW and CW chirality were used as initial states (see Fig. 1). In our simulation, a pulsed heat spot with Gaussian shape was introduced. The diameter of the heat spot (σ_d_) was varied from 40 to 100 nm. The room temperature and the maximum temperature of the heat spot (*T*_max_) were set to be 300 and 550 K. The rise and fall time of the heat spot was assumed to be zero to simplify the switching phenomena. The pulse length of the heat spot (*t*_p_) was varied from 1 to 20 ns and our simulation was continued up to 20 ns after the fall of the pulse. The material parameters *M*_s_, *K*_u_, *A*, and *D* of each prism were changed according to the temperature of each prisms. The temperature dependence of *M*_s_ was calculated by the mean field approximation^26^. *A* and *D* were assumed to be proportional to the cube of *M*_s_, and *K*_u_ was assumed to be proportional to the square of *M*_s_.

## Results

**Figure 2:**
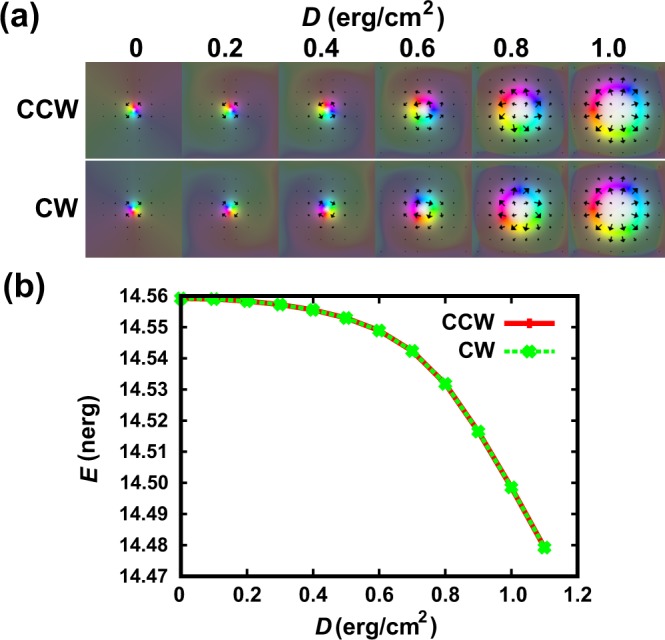
Effect of the *D* value on the Skyrmion. (**a**) Change of the structure of the magnetic moments and (**b**) change of the total magnetic energy by *D* for CCW and CW Skyrmions.

Before presenting the results of chirality switching, we show the relationship between *D* and the chirality of Skyrmion. Figure 2(a) shows the change of the magnetic moments of CCW and CW Skyrmions by *D*. In both cases, Néel-type Skyrmions appear for *D* = 1 erg/cm^2^. It indicates that the direction of the effective field induced by DMI is along the radial direction of the Skyrmion. It has no component perpendicular to the radial direction. This type of DMI does not affect the chirality of the Skyrmion. However Bloch-type Skyrmions with CCW and CW chiralities appear for *D* < 1 erg/cm^2^ cases. This finding shows that the origin of the chirality is not DMI but the demagnetizing field appearing in DW surrounding the Skyrmion. In these Skyrmions, the total magnetic energies for the same *D* value are the same as shown in Fig. 2(b).

**Figure 3:**
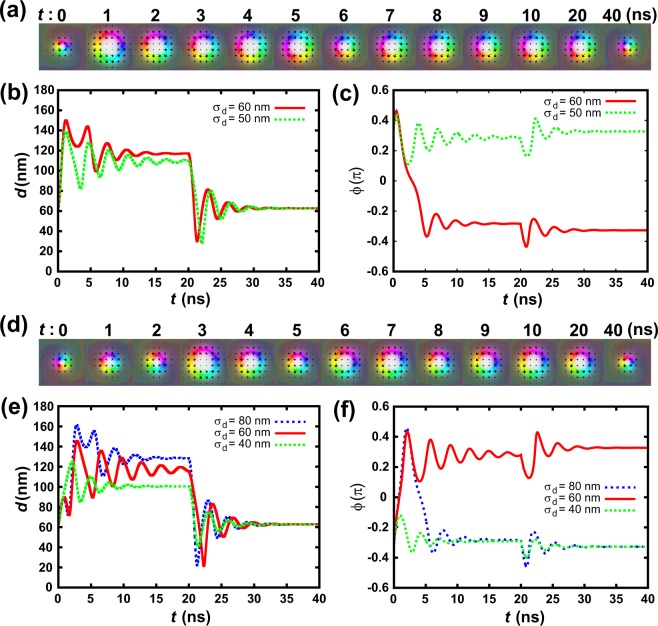
Simulated time-resolved chirality switching by a pulsed heat spot (σ_d_ = 60 nm and *t*_p_ = 20 ns) for (**a**–**c**) from CCW to CW and (**d**–**f**) from CW to CCW. (**a**,**d**) show the corresponding Skyrmion structures. (**b**,**e**) show the change of the Skyrmion radius with time. (**c**,**f**) show the change of the angle of the magnetic moments from the radial direction with time.

Figure 3 shows the switching of the chirality of the CCW and CW Skyrmions by introducing the pulsed heat spot. Here we have used the heat spot with σ_d_ = 60 nm and *t*_p_ = 20 ns. Figure 3(a) shows the simulated time-resolved chirality switching in the CCW Skyrmion by a pulsed heat spot. Figure 3(b,c) show the change of the diameter and the averaged angle of the magnetic moments from the radial direction of the Skyrmion with time. For σ_d_ = 60 nm case (red line), the diameter of the Skyrmion increases to *t* = 1.2 ns, and starts to breathe. The magnetic moments of the Skyrmion initially rotate to the CCW direction with the angle ϕ. However, the rotation direction switches at *t* = 0.45 ns with decreasing ϕ. The angle ϕ decreases continuously, and reaches −0.35π at *t* = 5 ns, where the chirality switched. In the first breathing in which the angle of the magnetic moments decreases continuously, the oscillation amplitude of the radius is small (~0.2π). The breathing starts again when the heat spot is stopped to be introduced. However, the amplitude of the oscillation is small and the associated chirality switching does not occur. Figure 3(b,c) also show no switching cases with small heat spot (σ_d_ = 50 nm). The breathing also starts but the angle does not reach 0, indicating it does not switch.

Figure 3(d) to (f) are the results for the switching from CW to CCW. For σ_d_ = 60 nm case (red line), the diameter of the Skyrmion increases slightly up to *d* = 89.8 nm with *t* = 0.87 ns. It further increases to 145.6 nm at *t* = 2.87 ns after one small breathing. For these periods, the magnetic moments rotate to the CCW direction with increasing ϕ, and it reaches 0.43π at *t* = 2.1 ns with switching the chirality. After that, breathing dominates. The breathing starts again when the heat spot is stopped to be introduced, however it does not switch in the same manner as the case from CCW to CW. With a small heat spot case (σ_d_ = 40 nm), the angle of the magnetic moments does not reach 0, and the chirality does not switch. With a large heat spot case (σ_d_ = 80 nm), the magnetic moments rotate to the CW direction after reaching the maximum, and it rotates back to the original direction. Thus, the chirality is found to be switched twice during the heating process, and it is not switched by the heat spot.

## Discussion

**Figure 4:**
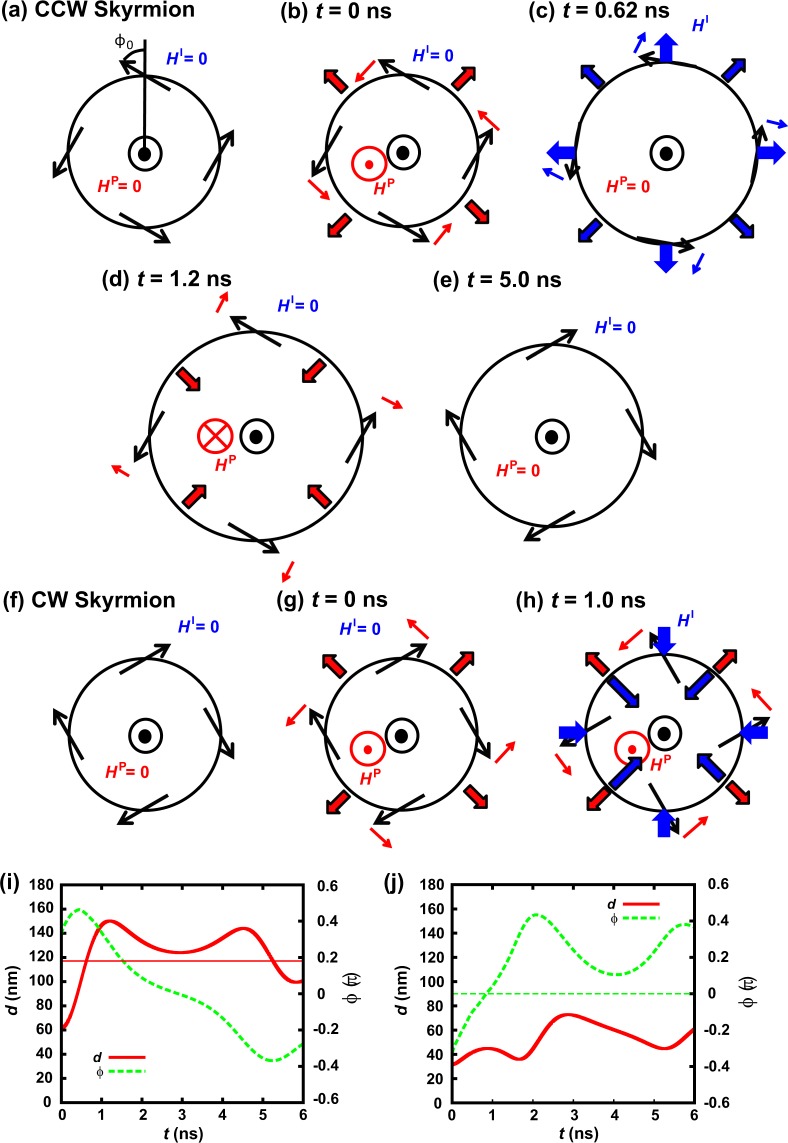
Chirality switching mechanism. The black circles and arrows show the central part of DW surrounding the Skyrmion and the magnetic moments at the center of DW, respectively. The blue thick arrows show the in-plane component of the effective field appearing in DW surrounding the Skyrmion (*H*^I^). The red circle shows the perpendicular component of the effective field appearing in DW (*H*^P^). Small blue and red arrows show the directions of the rotation of the magnetic moments at DW by *H*^I^ and *H*^P^, respectively. Thick blue and red arrows on black circles show the direction of the DW motion by *H*^I^ and *H*^P^, respectively. (**a**–**e**) Show the mechanisms to switch from CCW to CW. (**f**–**h**) Show the mechanisms to switch from CW to CCW. (**i**,**j**) Show the change of the Skyrmion radius and the angle of the magnetic moments up to 6 ns for CCW and CW Skyrmions, respectively.

In this way, the switching mechanism changes by the initial structure of the Skyrmion. In a CCW Skyrmion, it expands and then switches, but in a CW Skyrmion, it switches and then expands by a heat spot. The difference in the switching mechanism can be explained as follows. Figure 4(a) to (h) show the illustration of the magnetic moments on DW surrounding the Skyrmion, the effective fields acting on DW surrounding a Skyrmion, and the direction of the DW motion. Figure 4(i,j) show the change of the radius and the averaged angle of the magnetic moments of the Skyrmion with time up to 6 ns. Here, we take two fields into account: (i) the in-plane component of the effective field (*H*^I^) and (ii) the perpendicular component of the effective field (*H*^P^). They are the sum of the effective fields of the anisotropy, exchange and demagnetizing fields in addition to DMI. *H*^I^ changes depending on the angle of the magnetic moments in DW mainly. In the remanent state, *H*^I^ and *H*^P^ are zero (see Fig. 4(a,f)). The magnetic moments of DW are not parallel to DW by the effect of DMI. Suppose that the angle of the magnetic moments from the normal direction of DW is ϕ_0_. As discussed above, a Skyrmion expands by a heat spot, which is considered to be induced by *H*^P^ due to the application of the heat spot^17^.

For the CCW Skyrmion case, the magnetic moments rotate to the CCW direction by *H*^P^, and DW surrounding the Skyrmion moves to the direction to expand the Skyrmion diameter. (see Fig. 4(b)). This is the same mechanism of the DW motion by the perpendicular field. DW still moves even if the diameter reaches 117.2 nm with *t* = 0.62 ns as shown in Fig. 4(c,i), which is the diameter of the remanent state with σ_d_ = 60 nm of the heat spot (see Fig. 3(b) for *t* = 20 ns), by the effect of *H*^I^. In other words, DW moves continuously by the effect of DW mass, Döring mass^27^. This phenomenon is called overshoot^25^. When the diameter reaches the maximum at *t* = 1.2 ns (see Fig. 4(i) with *d*_max_ = 150 nm as appeared in Fig. 4(d)), *H*^P^ becomes negative because the diameter exceeds the size of the remanent state, 117.2 nm. The magnetic moments rotate to the CW direction by *H*^P^, and the chirality switches as shown in Fig. 4(e).

On the other hand, for the CW Skyrmion case (see Fig. 4(f)), the magnetic moments rotate to the CCW direction and the Skyrmion expands by *H*^P^ [see Fig. 4(g)). However, when the chirality switches, which means the angle of the magnetic moments exceeds zero, the Skyrmion shrinks by the effect of *H*^I^ (see Fig. 4(h)). This is the same mechanism of the backward motion of DW in a racetrack memory by Walker’s breakdown^25^. Here, the magnetic moments rotate continuously (see Fig. 4(h,j)). The Skyrmion expands again when the angle of the magnetic moments exceeds ϕ_0_, and the similar structure with Fig. 4(b) appears. Because the diameter is larger than that at *t* = 0 ns, the effective field acting on the Skyrmion is smaller than that at *t* = 0 ns. Hence, it cannot switch again. However, with the larger σ_d_ case, it expands further and switch back again in the same manner as CCW case. Supplementary Materials provide more details on the DW motion and Walker’s breakdown [see S3], Walker’s field in a thin film without and with the DMI cases [see S4], the relationship between the DW motion and the expansion of the Skyrmion [see S5], and the effective field acting on DW surrounding the Skyrmion by a heat spot [see S6]. In these materials, the switching mechanisms of the Skyrmion is explained not only qualitatively but also quantitatively.

**Figure 5:**
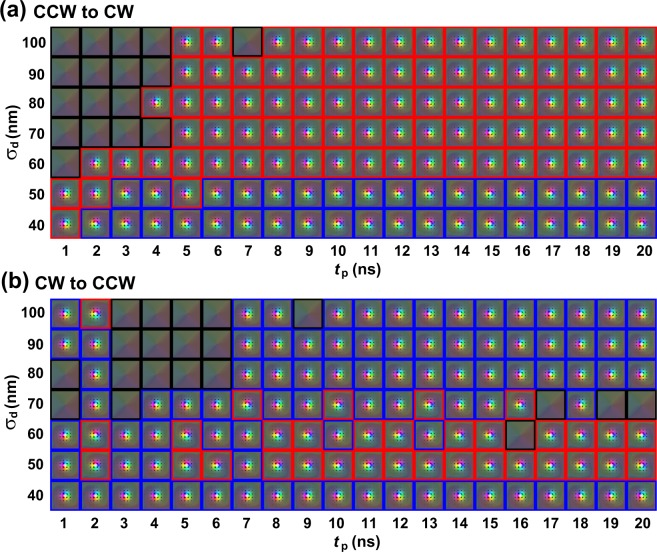
Diagrams plotting the final structures of the Skyrmion after the introduction of heat pulse on σ_d_ and *t*_p_ for the chirality switching (**a**) from CCW to CW and (**b**) from CW to CCW. Red, blue and black squares indicate three different cases of switching occurred, no switching occurred and Skyrmion annihilated, respectively.

Figure 5 shows the effects of the pulsed size and length on the chirality switching. Figure 5(a) shows the switching from CCW to CW. It switches by larger heat spot (σ_d_ $\underline{\underline{ > }}$ 60 nm) and longer pulse (*t*_p_ $\underline{\underline{ > }}$ 5 ns). As mentioned above, the Skyrmion should be expanded sufficiently to switch the chirality, followed by breathing, indicating larger *H*^P^ and longer pulse are required for coherent chirality switching. The Skyrmion disappears with a larger heat spot and a short pulse. The amplitude of the breathing increases with increasing heat pulse size. If the pulse stops to be introduced when the amplitude of the breathing is large, it shrinks vigorously and collapses.

Figure 5(b) shows the switching from CW to CCW. It switches by the heat spot of 50 $\underline{\underline{ < }}$ σ_d_ $\underline{\underline{ < }}$ 60 nm. The amount of the rotation is not enough for switching with a smaller heat spot than this σ_d_ range, and it switches twice with larger heat spot as mentioned before. Switching depends on the pulse length even with these sizes of the heat spot with shorter pulse below 8 ns. In not switching cases in these conditions, breathing still continues and it switches again when the pulse is stopped to be introduced. In these results, we assume the rise and fall time of the heat spot to be zero for the sake of argument. The effects of these time are shown in Supplemental Material S1. The effect of the misalignment, which is the distance between the Skyrmion center to the heat spot center, is also shown in Supplemental Material S2.

From these results, Skyrmions are found to switch to both ways, from CW to CCW and *vice versa*, with a larger heat spot of σ_d_ = 60 nm. Only the CW Skyrmion appears with the much larger heat spot of σ_d_ $\underline{\underline{ > }}$ 70 nm. Only the CCW Skyrmion appears with the larger heat spot of σ_d_ = 50 nm. By changing the heat spot size, chirality can be switched, which confirms that the heat-induced chirality switching is achieved reproducibly. In addition, a conventional thermal effect by a pulsed heat spot is considered as the case with a very large heat spot and long pulse length in our model^16,17^. For this case, the Skyrmion expands significantly and the chirality switches many times. Therefore the precise control of the heat spot size and the pulse length is required to control the chirality switching consistently.

In summary, we investigated the switching of the Skyrmion chirality dependent on the pulse heat spot size σ_d_ and the pulse heat time *t*_p_. We found that there are the optimized conditions for the Skyrmion chirality to be switched in both ways between CW and CCW only by heat pulse introduction, and that the changing of structure of the magnetic moments in Skyrmion during the pulse heating is different dependent on the initial Skyrmion chiral state. Such controllability can be used for a Skyrmion logic only by introducing heat.

## Supplementary information

Supplement text

## Data Availability

The datasets generated during the current study are available from the corresponding author on reasonable request.

## References

[1] Skyrme THR (1962). A unified field theory of mesons and baryons. Nucl. Phys.

[2] Röszler UK, Bogdanov AN, Pfleiderer C (2006). Spontaneous skyrmion ground states in magnetic metals. Nature.

[3] Muhlbauer S (2009). Skyrmion lattice in a chiral magnet. Science.

[4] Yu X (2010). Real-space observation of a two-dimensional skyrmion crystal. Nature.

[5] Jonietz F (2010). Spin transfer torques in MnSi at ultralow current densities. Science.

[6] Dzyaloshinsky I (1958). A thermodynamic theory of “weak” ferromagnetism of antiferromagnetics. J. Phys. Chem. Solids.

[7] Moriya T (1960). Anisotropic superexchange interaction and weak ferromagnetism. Phys. Rev..

[8] Yu XZ (2012). Skyrmion flow near room temperature in an ultralow current density. Nat. Commun..

[9] Iwasaki J, Mochizuki M, Nagaosa N (2013). Current-induced skyrmion dynamics in constricted geometries. Nat. Nanotech..

[10] Sampaio J, Cros V, Rohart S, Thiaville A, Fert A (2013). Nucleation, stability and current-induced motion of isolated magnetic skyrmions in nanostructures. Nat. Nanotech..

[11] Nagosa N, Tokura Y (2013). Topological properties and dynamics of magnetic skyrmions. Nat. Nanotech..

[12] Koshibae W (2015). Memory functions of magnetic skyrmions. Jpn. J. Appl. Phys..

[13] Tchoe Y, Han JH (2012). Skyrmion generation by current. Phys. Rev. B.

[14] Romming N (2013). Writing and deleting single magnetic skyrmion. Science.

[15] Jiang W (2015). Blowing magnetic skyrmion bubbles. Science.

[16] Finazzi M (2013). Laser-induced magnetic nanostructures with tunable topological properties. Phys. Rev. Lett..

[17] Koshibae W, Nagaosa N (2014). Creation of skyrmions and antiskyrmions by local heating. Nat. Commun..

[18] Nakatani Y, Hayashi M, Kanai S, Fukami S, Ohno H (2016). Electric field control of Skyrmions in magnetic nanodisks. Appl. Phys. Lett..

[19] Yu X (2012). Magnetic stripes and skyrmions with helicity reversals. Natl Acad. Sci. USA.

[20] Nakatani Y, Uesaka Y, Hayashi N (1989). Direct solution of the Landau-Lifshitz- Gilbert Equation for micromagnetics. Jpn. J. Appl. Phys..

[21] Nakatani Y, Thiaville A, Miltat J (2003). Faster magnetic walls in rough wires. Nature Material.

[22] Rohart S, Thiaville A (2013). Skyrmion confinement in ultrathin film nanostructures in the presence of Dzyaloshinskii-Moriya interaction. Phys. Rev. B.

[23] Endo M, Kanai S, Ikeda S, Matsukura F, Ohno H (2010). Electric-field effects on thickness dependent magnetic anisotropy of sputtered MgO/Co_40_Fe_40_B_20_/Ta structures. Appl. Phys. Lett..

[24] Kanai S (2013). In-plane magnetic field dependence of electric field-induced magnetization switching. Appl. Phys. Lett..

[25] Torrejon J (2014). H. Interface control of the magnetic chirality in CoFeB/MgO heterostructures with heavy-metal underlayers. Nat. Commun..

[26] Atxitia U (2007). Micromagnetc modeling of laser-induced magnetization dynamics using the Landau-Lifshitz-Bloch equation. Appl. Phys. Lett..

[27] Malozemoff, A. P. & Slonczewski, J. C. *Magnetic Domain Wall in Bubble Materials*, Academc Press, New York, 1979.

