# Supplementary material for: Switching of Skyrmion chirality by local heating
Source: Sci Rep. 2019 Sep 17;9:13475. doi: 10.1038/s41598-019-49875-7 (PMC6748957; doi:10.1038/s41598-019-49875-7)
Supplement: Supplementary file 1 — Supplement text [file 41598_2019_49875_MOESM1_ESM.docx]

**Switching of Skyrmion chirality by local heating**

Yoshinobu Nakatani, Keisuke Yamada, and Atsufumi Hirohata

**S1. THE RISE AND FALL TIME OF THE HEAT SPOT**

In the manuscript we used the heat spot with the rise and fall time to be zero. Here we investigate the effects of the rise and fall time of the heat spot.

(1) For the fall time (*t*_f_) of 1, 3, and 10 ns, Skyrmion behaviors with the pulse width of 1 to 8 ns were calculated to investigate the effect of *t*_f_ on the switching.

For the switching from CCW to CW, the switching condition does not change with *t*_f_ [see Figs. S1.1(a)-(c)]. These results can be explained as follows. A Skyrmion breathes when the heat pulse is introduced and stopped. When the pulse is introduced, the amplitude of breathing increases and the chirality switches. However when the pulse is not applied, the amplitude of breathing remains to be small and the chirality does not switch. Accordingly, when the pulse is introduced and stopped, the increased amplitude of breathing decreases with *t*_f_. Therefore the switching condition does not change with *t*_f._

For the switching from CW to CCW, the Skyrmion chirality switches with the larger heat pulse with increasing *t*_f_ [see Figs. S1.1(d)-(f)]. These results can be explained as follows. The CW chirality switches to the CCW state just after the application of the heat spot, followed by the expansion of the Skyrmion and breathing. If the short pulse or large pulse spot size is introduced, which is stopped before the completion of the breathing, the breathing is amplified and the Skyrion switches back again to the initial CW state in some cases. The effect of the termination of the heat pulse on the breathing decreases with increasing *t*_f_, resulting the switching to be difficult. Therefore the Skyrmion chirality switches only once even with the larger heat spot.

(2) For the raise time (*t*_r_) of 1, 3, and 10 ns for the switching from CCW to CW as well as *t*_r_ of 2 ns for the switching from CW to CCW, Skyrmion behaviors with the pulse width of 1 to 8 ns were calculated to investigate the effect of *t*_r_ on the switching.

For the switching from CCW to CW, the heat spot size required for switching increases with increasing *t*_r_ [see Figs. S1.2(a)-(c)]. Because the amplitude of the breathing decreases with increasing *t*_r_, the Skyrmion needs to be expanded to achieve the large amplitude of breathing for switching.

For the switching from CW to CCW, the heat spot size required for switching also increases with increasing *t*_r_. However less switching occurs and the chirality does not switch for *t*_r_ > 2 ns. As we mentioned above, for the switching from CW to CCW, it switches just after the introduction of the heat pulse. This is because the velocity of the Skyrmion expansion by the heat pulse decreases with increasing *t*_r_, and the velocity does not reach the critical value for switching.

From the above two cases, the effect of the fall time is very small for the switching. However the heat pulse size needs to be increased as the rise time is increased. In addition the rise time needs to be shorter than 2 ns for the switching from CW to CCW.

**S2. CONTROL OF THE HEAT SPOT POSITION**

In the manuscript, we assumed the heat spot is applied to the center of the Skyrmion. Here the effect of the misalignment, which is the distance between the Skyrmion center and the heat spot center, is investigated for the cases with the pulse width of 20 ns, the misalignment between 0 and 40 nm and the heat spot size between 50 and 90 nm.

For both CCW to CW (see Fig. S2.1) and CW to CCW (see Fig. S2.2) cases, the heat spot size for the switching is increased as the misalignment is increased. The heat gradient at DW surrounding the Skyrmion changes by the spot position with the misalignment. The larger heat spot is required to introduce large enough heat gradient for all the DW positions surrounding the Skyrmion. When the misalignment is 20 (0) nm, the heat spot size required for the switching is calculated to be 80 (60) nm for the CCW to CW case, and 60 (50) nm for the CW to CCW case, for example.

**S3. MAGNETIC FIELD INDUCED DOMAIN WALL MOTION AND WALKER’S BREAKDOWN**

We explain the mechanism of the Skyrmion chirality switching using the breakdown in the DW motion. Here we explain the mechanisms of the DW motion, and the breakdown by an external magnetic field [1]. We describe for the *D*=0 case first [S3], then explain the modification of the mechanisms by DMI [S4], and finally discuss the relationship between the DW motion and the expansion of the Skyrmion [S5].

In general, a Bloch wall, whose magnetization at the DW center is parallel to the wall, appears in a ferromagnetic thin film with perpendicular anisotropy [see Fig. S3.1(a)]. Because the perpendicular component of the magnetization to the wall plane [*x* direction in Fig. S3.1(a)] produces a demagnetizing field and increases the demagnetizing energy, the perpendicular component should become zero in the energy minimum state. The magnetization at the DW center is called the magnetization simply, and the azimuthal angle of the magnetization (φ) is defined from the wall normal direction, here after. There are two directions to the parallel direction of DW, indicating two types of the Bloch walls can be appeared [φ=±π/2 as shown in Figs. S3.1(b) and (c)]. As we mentioned above, the perpendicular component to the wall plane should be zero in the energy minimum state to reduce the demagnetizing field, and hence these magnetization directions are locked by the demagnetizing energy.

These Bloch walls move to the +*x* direction by a perpendicular external field (*H*_z_ > 0). The DW motion velocity is proportional to the field when the field is weak. However the velocity decreases and then increases slowly with a strong field. This mechanism can be explained as follows.

When the perpendicular field is applied to the Bloch wall, the magnetization rotates in the *x*-*y* plane by precessional motion, where the azimuthal angle increases, and the magnetization lies out of plane to the parallel direction of the wall [see Fig. S3.2(a)]. Because the negative perpendicular component of the magnetization to the wall plane (-*x* component) appears, the demagnetizing field to the +*x* direction appears, and the magnetization rotates in the *y-z* plane by this field [see Fig. S3.2(b)]. The polar angle of the magnetization decreases by the latter rotation and DW moves to the +*x* direction [see Fig. S3.2(c)]. When the external field is weak and the initial rotation effect is smaller than the latter effect by the demagnetizing field, the magnetization angle is determined by the angle, at which both effects balance, and DW moves with maintaining the magnetization angle. However if the external field is strong and the initial rotation effect is larger than that of the maximum value of the demagnetizing field, the magnetization rotates continuously in the *x*-*y* plane. In this case, the direction of the DW motion changes with time. For the case of π/2 < φ < π, the polar angle of the magnetization decreases with increasing the demagnetizing field and DW moves to the +*x* direction. However for the case of π < φ < 3π/2, the polar angle of the magnetization increases with the demagnetizing field and the DW moves to the –*x* direction as shown in Fig. S3.2(d). For the case of 3π/2 < φ < 2π, on the other hand, the direction of the DW motion changes back to the +*x*-direction as similarly observed for the former case.

Figure S3.3 summarizes the magnetization angle and the DW motion direction. The magnetic moment rotates along the CCW direction by the positive magnetic field (*H*_z_ > 0). DW moves to the +*x* direction when the magnetic moment is in the R2 and R4 regions in Fig. S3.3, and it moves to the –*x* direction when it is in the R1 and R3 regions. The average DW motion velocity decreases due to the change of the DW motion direction with time. This phenomenon is called Walker’s breakdown. When Walker’s breakdown occurs, the magnetization rotates continuously and the direction of the magnetization at the wall center switches continuously.

The critical field for Walker’s breakdown is called Walker’s field (*H*^W^). In the case of an infinitely thick film, *H*^W^ was estimated analytically as below because the maximum demagnetizing field can be obtained analytically.

*H*^W^ = 2 π*M_s_*α … (F1)

Here, *M_s_* is the saturation magnetization and α is the Gilbert damping constant. The field with the material used in this study with infinite thickness at *T* = 300 K is 1005 Oe. The field decreases for a finite thickness film, because the demagnetizing field decreases as the thickness is decreased. *H*^W^ with a finite thickness film can be obtained only by micromagnetic simulation.

The breakdown mechanism changes by DMI. Figures S3.4(a) and (b) show DW structures with weak DMI. In this case, the effective field with the +*x* direction is produced by DMI. If the DMI is strong, the magnetization aligns along the field, resulting in the case that φ becomes zero and a Néel wall appears. However with a weak DMI case, the Bloch like wall with a smaller magnetization angle appears. The relationship between the magnetization angle and the direction of the DW motion also changes by the DMI field.

Figure S3.5 shows this relationship. R2’ and R3’ increase because the direction of the DMI field is the same as that of the demagnetizing field in these regions. In R2’, the magnetization becomes hard to be rotated, and Walker’s field from R2’ to R3’ increases. On the other hand, R1’ and R4’ decrease because the direction of the DMI field is opposite to that of the demagnetizing field in these regions. In R4’, the magnetization becomes easy to be rotated, and Walker’s field from R4’ to R1’ decreases. In this way, there are two Walker’s fields existing for the weak DMI case. Because the latter field is weaker than the former field, the magnetization switches from R4’ to R1’ by a weak field, and DW moves with keeping the angle in R2’. The strong field is required to rotate the magnetization continuously.

**S4. WALKER’S FIELD IN THE THIN FILM WITHOUT AND WITH DMI CASES**

Next we obtain Walker’s fields with and without DMI for the cases of a thin film by simulation. We used a one-dimensional model with the same material parameters and the thickness used in this study, and Walker’s fields were obtained by the simulation of the DW motion [2].

Before to obtain the fields, we investigated the change of the magnetization angle by DMI on a Skyrmion and a DW.

Figure S4.1 shows the effect of DMI on the magnetization angle (φ) from the wall normal direction for the Skyrmion and DW with the material parameters at *T* = 300 K. In the case of DW, φ decreases and becomes zero at *D* = 0.2 erg/cm^2^. However in the case of the Skyrmion, φ becomes zero at *D* = 0.9 erg/cm^2^. This difference is due to the surface tension acting on DW surrounding the Skyrmion. For the Skyrmion, the magnetization on DW is pushed back by the tension. Accordingly the larger DMI value is required to reach the same angle. φ of the Skyrmion at *D* = 0.6 erg/cm^2^ is 1 rad. The DMI value for the same angle for DW is calculated to be 0.085 erg/cm^2^, Therefore we used this DMI value for the simulation of the DW motion.

At first we obtained Walker’s field at *D* = 0 erg/cm^2^ case. Figures 4.2(a) and (b) show the change of the DW motion velocity and the magnetization angle with time. The velocity increases with time and becomes constant when it reaches the saturation at *H* = 50 Oe (steady state motion) as shown in Fig. 4.2(a). Here the magnetization angle increases and also becomes constant [see Fig. 4.2(b)]. However the velocity oscillates and the angle increases with a stepwise manner with time at *H* = 55 Oe [see Figs. 4.2(a) and (b)]. It shows the magnetization switches continuously, indicating that Walker’s breakdown occurs. The angle increases by π in one breakdown. It shows the magnetization switches to the opposite direction in one breakdown. These figures confirm that Walker’s field decreases to about 55 Oe for a thin film with the thickness of 1.4 nm. Figure S4.2(c) shows the effects of the external field and the temperature on the averaged DW motion velocity. Walker’s field decreases as the temperature is increased, indicating that the saturation magnetization is decreased.

Next we obtained Walker’s field at *D* = 0.085 erg/cm^2^. Figures S4.3(a) and (b) show the change of the DW motion velocity and the magnetization angle with time. Here the initial magnetization angle is 1 rad [see Fig. S4.1]. The velocity increases with time and becomes constant when it reaches the saturation at *H* = 93 Oe (steady state motion) [see Fig. S4.3(a)]. The magnetization angle increases and also becomes constant as shown in Fig. S4.3(b). However the velocity oscillates and the angle increases with a stepwise manner with time at *H* = 99 Oe [see Figs. S4.3(a) and (b)]. These results show the magnetization switches continuously, indicating that Walker’s breakdown occurs. Unlike at *D* = 0 erg/cm^2^, two successive negative peaks appear in Fig S4.3(a), and the step width is calculated to be 2π in Fig S4.3(b). These figures show that two breakdowns occur successively, and the magnetization rotates 2π in one breakdown. Figure S4.3(c) shows the effect of the external field and the temperature on the averaged DW motion velocity. Walker’s field is increased by DMI. Note that Walker’s field in this figure is the stronger one.

As mentioned above, the magnetization rotates once in the case of weaker Walker’s field, which cannot be obtained from the averaged DW motion velocity. We have to check the magnetization switching in the DW motion with the initial magnetization angle in R4’ to obtain the critical field.

Figures S4.3(d) and (e) represent the case with the initial magnetization angle of -1 rad. The DW motion velocity decreases and the magnetization angle switches from -1 to 1 rad at *t* = 42 ns with the field of 18 Oe (weaker Walker’s field).

Figure S4.4 shows the change of Walker’s field with temperature for the *D* = 0 and 0.085 erg/cm^2^ cases. *H*_w1_ and *H*_w2_ show that the stronger and weaker fields, respectively. Walker’s field at *D* = 0 and *H*_w1_ are calculated to be about 50 and 80 Oe, respectively, both of which decrease with temperature. However *H*_w2_ is about 20 Oe, and does not change with temperature.

**S5. RELATION OF THE DOMAIN WALL MOTION AND EXPANTION OF THE SKYRMION**

Figures S5.1(a) to (d) summarize the magnetizations of DWs and Skyrmions. The magnetization angles in Fig. S5.1(a) [S5.1(b)] are similar with the angles in Fig. S5.1(c) [S5.1(d)]. The switching mechanisms of each Skyrmion can be explained by the mechanisms of Walker’s breakdown for each DW.

In the case of the CW Skyrmion, it switches when it expands. The magnetizations before and after switching are shown with black and red arrows in Fig. S5.2(a). The switching mechanism is the same as that of the Bloch wall in Fig. S5.2(b). This switching is the case from R4’ to R1’ by the weaker Walker’s field, which is about 20 Oe in this study.

In the case of the CCW Skyrmion, the magnetization angle increases but it does not switch when it expands [see Fig. S5.3(a)]. Because it corresponds with the case in Fig. 5.3(b), the stronger Walker’s field (about 80 Oe) is required for switching. The fact that the CCW Skyrmion does not switch when it expands means the effective field produced by temperature is smaller than 80 Oe. The CCW Skyrmion switches when it shrinks. In this case the direction of the effective field switches. This switching corresponds to the case from R1’ to R4’ by the weak Walker’s field (about 20 Oe) as shown in Fig. S5.3(c).

**S6. EFFECTIVE FIELD ON THE SKYRMION BY HEAT SPOT**

Here we calculate the effective field acting on DW surrounding a Skyrmion induced by a heat spot. The temperature of the film increases by a heat spot. The material parameters decrease as the temperature is increased, and the effective field acting on DW surrounding a Skyrmion changes. Because DW moves by the perpendicular component of the field mainly, we investigate this component.

Figure S6.1(a) shows the effective field in the radial direction of a CW Skyrmion, showing the simulated results of the field without a heat pulse and just after the application of a heat pulse of σ_d_=60 nm . Both fields are almost the same especially at the center region of DW. DW does not move without a heat pulse, and therefore the difference in the fields is the effective field generated by a heat pulse. Figure S6.1(b) shows the difference in the fields. The effective field acting on the center region of DW is about 50 Oe, which is between *H*_W1_ and *H*_W2_ in Figure S4.4. It confirms that the CW Skyrmion can be switched by the field. The effective field acting on the CCW Skyrmion is the same as the value as shown in these figures. The switching field is smaller than *H*_W1_. The CCW Skyrmion, on the other hand, cannot be switched by the field, The Skyrmion only expands by the heat pulse keeping the chirality.

--------------------------------------------------------------------------------------------------------------------

[1] Malozemoff, A. P. & Slonczewski, J. C. *Magnetic Domain Wall in Bubble Materials*, Academc Press, New York, 1979.

[2] Thiaville, A., Rohart, S., Jue, E., Cros, V. & Fert, A., Dynamics of Dzyaloshinskii domain walls in ultrathin magnetic films, *Euro. Phys. Lett.*, 100, 57002 (2012).

FIG. S1.1 Effect of the fall time on switching. The diameter of the heat spot is varied from 40 to 70 nm. The pulse length is varied from 1 to 8 ns. The fall time of 1, 3, and 10 ns are calculated. (a) to (c) show the case from CCW to CW. (d) to (f) show the case from CW to CCW.

FIG. S1.2 Effect of the rise time on switching. The diameter of the heat spot is varied from 40 to 70 nm. The pulse length is varied from 1 to 8 ns. The rise time of 1, 3, and 10 ns are calculated for the case from CCW to CW [(a) to (c)]. The rise time of 1 and 2 ns are calculated for the case from CW to CCW [(d) and (e)].

FIG. S2.1 Effect of the misalignment of a Skyrmion and a heat spot for the case from CCW to CW. The amount of misalignment is varied from 0 to 40 nm and the diameter of the heat spot is varied from 50 to 90 nm.

FIG. S2.2 Effect of the misalignment of a Skyrmion and a heat spot for the case from CW to CCW. The amount of misalignment is varied from 0 to 40nm and the diameter of the heat spot is varied from 50 to 90 nm.

FIG. S3.1 Magnetization distribution of a Bloch wall for *D* = 0 erg/cm^2^, showing (a) the Bloch wall, (b) and (c) the magnetization directions at the center region of DW.

FIG. S3.2 DW motion mechanism by an external perpendicular field. (a) The magnetization rotates in the *x*-*y* plane to the CCW configuration by the field. (b) The –*x* component of the magnetization produces a demagnetizing field along the +*x* direction. The magnetization rotates in the *y*-*z* plane by the demagnetizing field. (c) DW moves to the +*x* direction. (d) For a very strong external field, the magnetization rotates more in the *x*-*y* plane and rotates in the *y*-*z* plane to the opposite direction. (e) DW moves along the –*x* direction.

FIG. S3.3 Change of the DW motion direction by the strong field at *D* = 0 erg/cm^2^. The magnetization rotates continuously with the strong field. The direction of the DW motion changes by the direction of the magnetization.

FIG. S3.4 Magnetization distribution in a Bloch wall at *D* > 0 erg/cm^2^. (a) and (b) Magnetization directions at the center region of DW.

FIG. S3.5 Change of the DW motion direction by the strong field at *D* > 0 erg/cm^2^. The magnetization angle to change the direction of the DW motion changes by DMI.

FIG. S4.1 Change of the magnetization angle by DMI for a DW and a Skyrmion. The magnetization angle changes with small *D* in the DW case.

FIG. S4.2 Time resolved DW motion at *D* = 0 erg/cm^2^. (a) Change of the velocity with time. (b) Change of the magnetization angle with time. (c) Effect of the external field and the temperature on the averaged DW motion velocity.

FIG. S4.3 Time resolved DW motion at *D* > 0 erg/cm^2^. (a) Change of the velocity with time. (b) Change of the magnetization angle with time. (c) Effect of the external field and the temperature on the averaged DW motion velocity. The thick and thin lines show the cases at *D*=0.085 and 0 erg/cm^2^, respectively. (d) Change of the velocity with time. (e) Change of the magnetization angle with time.

FIG. S4.4 Change of Walker’s field at *D* = 0 and *D* > 0 erg/cm^2^ with respect to temperature.

FIG. S5.1 The magnetization distribution in a DW and a Skyrmion. The magnetization distribution in the Bloch wall with φ < π/2 corresponds to the CCW Skyrmion [(a) and (c)]. The magnetization distribution in the Bloch wall with φ > -π /2 corresponds to the CW Skyrmion [((b) and (d)].

FIG. S5.2 Chirality switching of a CW Skyrmion. (a) The switching mechanism of a CW Skyrmion is similar to (b) the breakdown of the Bloch wall with φ > -π /2.

FIG. S5.3 Chirality switching of a CCW Skyrmion. (a) The switching mechanism of a CCW Skyrmion is similar to (b) the breakdown of the Bloch wall with φ < π/2. A very strong effective field is required for the switching. (c) The direction of the effective field switches when the Skyrmion shrinks. The switching mechanism corresponds to the breakdown of the Bloch wall with φ > -π /2.

FIG. S6 Effective field on the Skyrmion by the heat spot. (a) The effective field in the radial direction of a CW Skyrmion. (b) The difference in the effective fields.
